# Analysis of Clinical Characteristics and Poor Prognostic Predictors in Patients With an Initial Diagnosis of Autoimmune Encephalitis

**DOI:** 10.3389/fimmu.2019.01286

**Published:** 2019-06-07

**Authors:** Xiaowei Qiu, Haiqing Zhang, Dongxu Li, Jing Wang, Zhigang Jiang, Yuanzhong Zhou, Ping Xu, Jun Zhang, Zhanhui Feng, Changyin Yu, Zucai Xu

**Affiliations:** ^1^Department of Neurology, Affiliated Hospital of Zunyi Medical University, Guizhou, China; ^2^Key Laboratory of Brain Science, Zunyi Medical University, Guizhou, China; ^3^Department of Neurology, The First Affiliated Hospital of Chongqing Medical University, Chongqing, China; ^4^Department of Preventive Health Care, Affiliated Hospital of Zunyi Medical University, Guizhou, China; ^5^School of Public Health, Zunyi Medical University, Guizhou, China; ^6^Department of Neurology, Affiliated Hospital of Guizhou Medical University, Guizhou, China

**Keywords:** autoimmune encephalitis, predictor, neutrophil-to-lymphocyte ratio, immunotherapy, modified Rankin Scale, prognosis

## Abstract

**Purpose:** We aimed to retrospectively analyze the clinical features, laboratory and imaging results, and predictors of poor prognosis for patients with an initial diagnosis of autoimmune encephalitis (AE) at the Affiliated Hospital of Zunyi Medical University.

**Methods:** Fifty patients with an initial diagnosis of AE who were admitted to our hospital from May 2014 to May 2018 were enrolled retrospectively. Clinical characteristics and experimental test data, including the neutrophil-to-lymphocyte ratio (NLR), were collected from medical records within 24 h of admission. Independent prognostic factors were determined by multivariate logistic regression analysis. A good or poor prognosis for patients was defined based on the modified Rankin Scale (mRS). The correlation between the immunotherapy latency and prognostic mRS score was determined using the Spearman rank correlation test.

**Results:** Univariate analysis indicated that increased NLR (*P* = 0.001), decreased lymphocyte counts (*P* = 0.001), low serum albumin (*P* = 0.017), consciousness disorders (*P* = 0.001), epileptic seizures (*P* = 0.007), extrapyramidal symptoms (*P* = 0.042), abnormal electroencephalogram (EEG) findings (*P* = 0.001), abnormal brain magnetic resonance imaging (MRI) findings (*P* = 0.003), and pulmonary infection complications (*P* = 0.000) were associated with the poor prognosis of AE. Multivariate logistic regression analysis showed that NLR (odds ratio [OR] 2.169, 95% confidence interval [CI] 1.029–4.570; *P* < 0.05) was an independent risk factor for predicting the poor prognosis of AE. NLR > 4.45 was suggested as the cut-off threshold for predicting the adverse outcomes of AE. In addition, we revealed that there was a positive correlation between immunotherapy latency and mRS score (r_s_ = 0.535, *P* < 0.05).

**Conclusions:** NLR may have predictive value for the poor outcomes of AE. Early initiation of immunotherapy is associated with a good prognosis.

## Introduction

Autoimmune encephalitis (AE) is a severe inflammatory disorder of the brain that is mediated by autoimmune mechanisms and characterized by prominent neuropsychiatric symptoms. AE, which is thought to be associated with antibodies against neuronal cell-surface proteins, ion channels, or receptors (1), accounts for about 20% of all adult encephalitis cases (2). Typical clinical manifestations include epileptic seizures, psychiatric and behavioral disorders, decreased levels of consciousness, memory and cognitive impairment, extrapyramidal symptoms, and central hypoventilation (3, 4). Since the discovery of anti-N-methyl-D-aspartate receptor (anti-NMDAR) antibodies by Dalmau et al. (5), more than a dozen new types of autoantibodies have been identified (6). Anti-NMDAR encephalitis is the most common type of AE, followed by anti-leucine-rich glioma-inactivated 1 (anti-LGI1) encephalitis (7) and anti-γ-aminobutyric acid B receptor (anti-GABA_B_R) encephalitis. Other types of antibodies include anti-contactin-associated protein-like 2 (anti-CASPR2) antibody and anti-α-amino-3-hydroxy-5-methyl-4-isoxazole propionate receptor (anti-AMPAR) antibody. The presence of corresponding autoantibodies contributes to diagnosis; however, because existing criteria for AE rely on antibody testing and the response to immunotherapy, delays in diagnosis, and missed diagnosis of antibody-negative patients can occur (8). A clinical approach to the diagnosis of AE was put forward jointly by international experts, providing a basis for the early diagnosis of this disease (8). In addition, AE is a severe neurological disorder that is characterized by complicated clinical manifestations and frequent complications. Some cases are associated with tumors. Immunotherapy, intensive care unit (ICU) support, and multidisciplinary treatments can be combined to mitigate the disease (9). At present, the efficacy of immunotherapy and factors that affect patients' poor prognosis have not been determined. Thus, research on the prognostic factors of AE has great clinical and social significance.

AE is recognized as a chronic autoimmune disease characterized by the presence of antigen-specific antibodies in serum and cerebrospinal fluid (CSF) resulting from dysfunction of the immune system regulation and persistent inflammation (10). The neutrophil-to-lymphocyte ratio (NLR) is a commonly used and very significant systemic inflammation biomarker. NLR is calculated as the absolute count of neutrophils divided by the absolute count of lymphocytes (11). Moreover, NLR has been suggested as a marker for the general immune response to various stress stimuli. Prior studies have shown that increased NLR is a prognostic marker in patients with various cancers, including pancreatic cancer, lung cancer, gastric cancer, hepatocellular carcinoma, prostate cancer, and malignant mesothelioma (12–16). In addition, several reports have demonstrated that altered NLR has prognostic value in diabetes mellitus, hypertension, acute myocardial infarction, cerebrovascular disease, peripheral arterial disease, and chronic kidney disease (17–20). Recent studies have also shown that an abnormal NLR level is associated with some autoimmune diseases (21, 22). However, to our knowledge, the relationship between NLR and AE has not been studied so far. Therefore, in this study, we evaluated the association between NLR and prognosis in AE patients and whether NLR is an independent risk factor for predicting the poor prognosis of AE.

## Methods

### Research Subjects

This retrospective study complied with the recommendations of the Ethics Committee of Affiliated Hospital of Zunyi Medical University. The protocol was approved by the Ethics Committee of Affiliated Hospital of Zunyi Medical University. All patients or their relatives were informed of the study and signed written informed consent in accordance with the Declaration of Helsinki. We reviewed all the medical records of patients with an initial diagnosis of AE admitted to the Department of Neurology, Affiliated Hospital of Zunyi Medical University, from May 2014 to May 2018. We reassessed the diagnosis basis and followed up with patients by telephone every 3 months after discharge. The inclusion criteria were based on the clinical diagnostic criteria for AE suggested by Mittal and Graus in 2016. Patients were categorized as “definite,” “probable,” or “possible” according to the adapted criteria (8). The diagnostic criteria for the “definite” group were the detection of antibodies against neuronal membrane or synaptic proteins in CSF and/or serum. Autoantibody-negative but “probable” AE did not meet the diagnostic criteria of the “definite” group but fulfilled all four other criteria supporting AE. Correspondingly, the following exclusion criteria were considered: other acute neurological diseases found during follow-up; not meeting the clinical diagnostic criteria for AE; loss to follow-up; other autoimmune diseases; and incomplete clinical data.

### Data Collection

The following basic clinical data were collected: age at onset, sex, clinical manifestations, interval from onset to admission, immunotherapy latency (the time interval from onset to the initiation of immunotherapy), prodromal symptoms, pulmonary infection complications, treatment methods, and hospital stay. In addition, cranial magnetic resonance imaging (MRI) findings, electroencephalogram (EEG) data, laboratory tests, CSF examination (pressure, white blood cell [WBC] counts, and protein, glucose and chloride levels), and autoantibody tests of serum and CSF were reviewed from medical records and electronic databases. The laboratory tests included the following: WBC counts, neutrophil counts, lymphocyte counts, platelet counts, NLR, and the levels of hemoglobin, sodium (Na), potassium (K), chlorine (Cl), calcium (Ca), and albumin. These experimental examinations were recorded within 24 h of admission. NLR was defined as a simple ratio between the absolute neutrophil count and the absolute lymphocyte count. Laboratory tests except NLR were divided into low, normal, and high values based on reference intervals.

Based on previous reports on AE (3), the main symptoms were divided into the following categories: consciousness disorders; epileptic seizures; mental and psychiatric and behavior disorders; and extrapyramidal symptoms. The inflammatory CSF needed to meet at least 2 of the following criteria: an increase in the number of CSF cells (≥5 leukocytes/mm^3^), an increase in the rate of immunoglobulin G (IgG) synthesis, or the appearance of CSF-specific oligoclonal bands. Supportive cranial MRI included T2-weighted fluid-attenuated inversion recovery (FLAIR) hyperintensity on one or both sides of the mesial temporal lobes, multiple inflammatory lesions, or demyelination involving gray and white matter. Supportive EEG included abnormal slow-wave activity and epileptiform discharges (8). Patient serum and CSF samples were simultaneously obtained and sent to Beijing Kindstar Global Company for testing.

### Disease Prognosis Evaluation

The modified Rankin Scale (mRS) was used to evaluate neurological function at the time of admission, at discharge from the hospital, and during the follow-up period. The mRS score includes 6 categories (23, 24): if patients had a full recovery (mRS 0 point); if patients had no significant functional impairment and were able to complete all daily duties and activity despite some symptoms (mRS 1 point); if patients had mild-moderate disability and were unable to complete all previous activities but could independently take care of their own affairs (mRS 2–3 points); if patients had severe disability and required others to take care of them (mRS 4 points); if patients had severe disability and required intensive care (mRS 5 points); and death (mRS 6 points). According to the mRS during the follow-up period, we divided all patients into two groups: patients with an mRS score of 0–1 were defined as “good prognosis”; patients with an mRS score of 2–6 were defined as “poor prognosis.”

### Statistical Analysis

All statistical analyses were performed using SPSS statistical software (version 22.0). Measurement data were presented in the form of “mean ± standard deviation” and/or “median (range),” whereas count data were presented as number (percentage). Univariate analysis was performed to compare the differences between the two groups. Independent Student's *t*-test was used for normally distributed variables, while the Mann-Whitney test was used for non-normally distributed variables. Categorical variables were compared using the chi-squared test. Logistic regression analysis was performed to determine the independent predictors of poor prognosis. The correlation between the immunotherapy latency and prognostic mRS score was determined using the Spearman rank correlation test. The optimal cutoff value for the NLR to serve as a prognostic marker for AE was determined from receiver operating curve (ROC) analysis. *P*-values < 0.05 (two-sided) were considered statistically significant.

## Results

### Patient Profile

**Figure 1:**
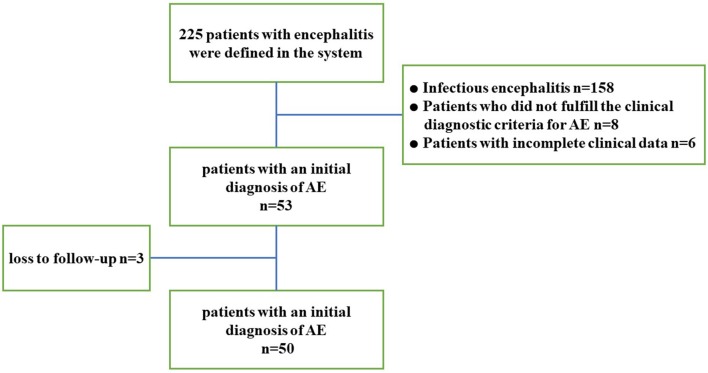
Study flowchart. AE, autoimmune encephalitis.

The search of the electronic database resulted in 225 potential encephalitis cases. A total of 50 patients with AE were included in the study (Figure 1 provides the flowchart of patient selection). Nine cases with positive antibodies were considered “definite AE,” including 7 patients positive for anti-NMDAR antibody, 1 patient positive for anti-GABA_B_R antibody, and 1 patient positive for anti-AMPAR antibody. Sixteen cases negative for antibodies were considered “probable AE,” and 25 cases were categorized as “possible AE.” All patients showed acute or subacute onset, and 33 (66%) exhibited prodromal symptoms such as headache and other clinical symptoms of non-specific upper respiratory tract infection symptoms. The average time from onset to admission was 10 days. Thirty-nine patients (78%) were initially misdiagnosed with viral encephalitis, psychosis, cerebrovascular disease, or other diseases. Among these patients, 2 had lung tumors, 1 had thymoma, and 1 had multiple myeloma. During the entire course of the disease, 19 patients (38%) developed fever, 9 patients (18%) had central hypoventilation, 13 patients (26%) had pulmonary infection complications, and 4 (8%) had been treated in the ICU. One patient died of small cell lung cancer during follow-up. The clinical characteristics and demographic information of the subjects are summarized in Table 1.

**Table 1:** Characteristics of the study population (*n* = 50).

**Characteristics**	**Patients (%)**
Sex (male/female)	31/19
Age mean, range (years)	39,14–74
Prodromal symptoms	33 (66%)
Interval between onset and Hospitalization mean, range (days)	10,1–60
Fever	19 (38%)
**Initial symptoms**	
Consciousness disorders	8 (16%)
Epileptic seizures	16 (32%)
Psychiatric and behavior disorders	19 (38%)
Extrapyramidal symptoms	3 (6%)
Other	4 (8%)
Consciousness disorders	25 (50%)
Epileptic seizures	25 (50%)
Psychiatric and behavior disorders	34 (68%)
Extrapyramidal symptoms	17 (34%)
Speech disturbances	5 (10%)
Memory deficits	7 (14%)
Autonomic dysfunction	1 (2%)
Mechanical ventilation	9 (18%)
Abnormal EEG results	33 (66%)
Abnormal brain MRI results	21 (42%)
Increased CSF pressure	9 (18%)
Increased CSF protein	29 (58%)
Increased CSF WBC counts	21 (42%)
Neutrophil count (10^9^ /L) (median IQR)	5.30 (3.73–8.20)
Lymphocyte count (10^9^ /L) (median IQR)	1.66 (1.14–2.03)
NLR (median IQR)	3.72 (2.16–5.56)
Pulmonary infection complications	13 (26%)
Tumor	4 (8%)
Immunotherapy	20 (40%)
Average hospital stay, range (days)	22.5, 5–99

*IQR, interquartile range; EEG, electroencephalogram; MRI, magnetic resonance imaging; CSF, cerebral spinal fluid; NLR, neutrophil-to-lymphocyte ratio*.

### Auxiliary Examinations

The brain MRI, EEG, and CSF results of all patients were available. EEG findings were abnormal in 33 patients (66%), including 10 patients with epileptiform discharges (such as spike waves, sharp waves, spike slow wave complex, or sharp slow wave complex), 22 patients with unilateral or bilateral non-specific slow waves, and 1 patient with δ brushes. Brain MRI findings showed that the lesions were located in the frontal lobes, temporal lobes, parietal lobes, occipital lobes, insular lobes, hippocampus, basal ganglia, thalamus, cerebellum, cortex, and white matter. Twenty-one patients (42%) had specific T2-signal hyperintensities. These affected brain regions mainly included the medial temporal lobes, frontal and parietal lobes, and/or subcortical regions. Non-specific changes/demyelinating lesions were present in 13 patients (26%), whereas 16 patients had no abnormalities (32%). CSF findings revealed that 21 patients (42%) displayed pleocytosis, and 29 patients (58%) had high concentrations of total protein.

### Treatment and Outcome

Twenty (40%) patients received immunotherapy, including eight patients with methylprednisolone (intravenous infusion, 1 g/day; 5 days); two patients with immunoglobulin (intravenous infusions, 0.4 g/kg; 5 days); nine patients with a combination treatment of IVIg and intravenous methylprednisolone; and one patient with a combination therapy of plasma exchange, IVIg, and intravenous methylprednisolone. None of our patients received second-line therapy (rituximab, cyclophosphamide, or other) due to medical insurance restrictions or drug side effects. The median follow-up time was 11 months (8–27 months). At the end of the follow-up period, 33 patients (66%) attained a good prognosis, whereas 17 patients (34%) had poor prognosis. Among all patients, 33 patients (66%) had mRS scores of 0 or 1. Meanwhile, 8 patients (16%) had mRS scores of 2, and 4 patients (8%) had mRS scores of 3. Additionally, 2 patients (4%) reached 4 points, and 2 patients (4%) received 5 points. Unfortunately, 1 patient (2%) died by the end of the study (mRS 6). Three patients relapsed during follow-up. Two patients with anti-NMDAR encephalitis also achieved a good prognosis without immunotherapy.

### Predictors of Prognosis

Univariate analysis indicated that there were significant differences between the good and poor outcome groups in laboratory values, including the NLR (*P* = 0.001), lymphocyte counts (*P* = 0.001), and albumin (*P* = 0.017). We found that the median NLR was significantly higher in the poor prognosis group than in the good prognosis group. In addition, consciousness disorders (*P* = 0.001), epileptic seizures (*P* = 0.007), extrapyramidal symptoms (*P* = 0.042), abnormal EEG findings (*P* = 0.001), abnormal MRI findings (*P* = 0.003), and pulmonary infection complications (*P* = 0.000) were associated with worse prognosis of AE (Table 2).

**Table 2:** Univariate analysis of prognostic factors associated with AE.

**Variables**	**Good prognosis (*n* = 33)**	**Poor prognosis (*n* = 17)**	***P*-value**
Age (years), (mean ± SD)	39.06 ± 17.74	38.06 ± 19.33	0.855
**Sex**			
Male	19 (57.6%)	12 (70.6%)	0.369
Female	14 (42.4%)	5 (29.4%)	
**Duration from onset to admission**			
≤ 2 wk	26 (78.8%)	13 (76.5%)	0.851
>2 wk	7 (21.2%)	4 (23.5%)	
**Fever**			
≤ 37.5°C	21 (63.6%)	10 (58.8%)	0.740
>37.5°C	12 (36.4%)	7 (41.2%)	
**Consciousness disorders**			
Yes	11 (33.3%)	14 (82.4%)	**0.001**
No	22 (66.7%)	3 (17.6%)	
**Epileptic seizures**			
Yes	12 (36.4%)	13 (76.5%)	**0.007**
No	21 (63.6%)	4 (23.5%)	
**Psychiatric and behavior disorders**			
Yes	22 (66.7%)	12 (70.6%)	0.778
No	11 (33.3%)	5 (29.4%)	
**Extrapyramidal symptoms**			
Yes	8 (24.2%)	9 (52.9%)	**0.042**
No	25 (75.8%)	8 (47.1%)	
**Brain MRI results**			
Abnormal	9 (27.3%)	12 (70.6%)	**0.003**
Normal	24 (72.7%)	5 (29.4%)	
**EEG results**			
Abnormal	17 (48.5%)	16 (94.1%)	**0.001**
Normal	16 (51.5%)	1 (5.9%)	
**CSF pressure, mmH**_**2**_**O**			
≥230	5 (15.2%)	4 (23.5%)	0.465
< 230	28 (84.8%)	13 (76.5%)	
**CSF WBC count**			
Normal	19 (57.6%)	10 (58.8%)	0.933
High	14 (42.4%)	7 (41.2%)	
**CSF protein level, mg/L**			
≤ 400	13 (39.4%)	8 (47.1%)	0.603
>400	20 (60.6%)	9 (52.9%)	
**CSF glucose level**			
Low	1 (3.0%)	1 (5.9%)	0.655
Normal	27 (81.8%)	12 (70.6%)	
High	5 (15.2%)	4 (23.5%)	
**CSF chloride level**			
Low	1 (3.0%)	1 (5.9%)	0.830
Normal	29 (87.9%)	15 (88.2%)	
High	3 (9.1%)	1 (5.9%)	
**Blood potassium level**			
Low	5 (15.2%)	5 (29.4%)	0.232
Normal	28 (84.8%)	12 (70.6%)	
**Blood sodium level**			
Low	6 (18.2%)	5 (29.4%)	0.221
Normal	27 (81.8%)	11 (64.7%)	
High	0 (0.0%)	1 (5.9%)	
**Blood chlorine level**			
Low	2 (6.1%)	2 (11.8%)	0.277
Normal	31 (93.9%)	14 (82.4%)	
High	0 (0%)	1 (5.9%)	
**Blood calcium level**			
Low	12 (36.4%)	8 (47.1%)	0.465
Normal	21 (63.6%)	9 (52.9%)	
**Albumin**			
Low	18 (54.5%)	15 (88.2%)	**0.017**
Normal	15 (45.5%)	3 (11.8%)	
**WBC count**			
Normal	26 (78.8%)	12 (70.6%)	0.520
High	7 (21.2%)	5 (29.4%)	
**Neutrophil count**			
Normal	24 (72.7%)	9 (52.9%)	0.162
High	9 (27.3%)	8 (47.1%)	
**Lymphocyte count**			
Low	2 (6.1%)	8 (47.1%)	**0.001**
Normal	31 (93.9%)	9 (52.9%)	
**Hemoglobin**			
Low	10 (30.3%)	7 (41.2%)	0.603
Normal	22 (66.7%)	10 (58.8%)	
High	1 (3.0%)	0 (0%)	
**Platelet count**			
Low	1 (3%)	2 (11.8%)	0.203
Normal	22 (66.7%)	13 (76.5%)	
High	10 (30.3%)	2 (11.8%)	
NLR (median IQR)	2.92 (1.87–4.01)	5.60(4.56–11.49)	**0.001**
**Mechanical ventilation**			
Yes	4 (12.1%)	5 (29.4%)	0.236
No	29 (87.9%)	12 (70.6%)	
**Pulmonary infection complications**			
Yes	3 (9.1%)	10 (58.8%)	**0.000**
No	30 (90.9%)	7 (41.2%)	

*AE, autoimmune encephalitis; SD, standard deviation; IQR, interquartile range; EEG, electroencephalogram; MRI, magnetic resonance imaging; CSF, cerebral spinal fluid; WBC, white blood cell; NLR, neutrophil-to-lymphocyte ratio. Reference interval: CSF WBC count: 0–5 × 10^6^/L; CSF protein level, 200–400 mg/L; CSF glucose level, 2.5–4.4 mmol/L; CSF chloride level, 120–130 mmol/L; blood potassium level, 3.5–5.3 mmol/L; blood sodium level, 137–147 mmol/L; blood chlorine level, 99–110 mmol/L; blood calcium level, 2.20–2.65 mmol/L; albumin, 40–55 g/L; WBC count, 3.5–10 × 10^9^/L; neutrophil count, 1.8–6.3 × 10^9^/L; lymphocyte count, 1.1–3.2 × 10^9^/L; hemoglobin, 115–150 g/L; platelet count, 101–320 × 10^9^/L. P values < 0.05 are considered statistically significant*.

**Figure 2:**
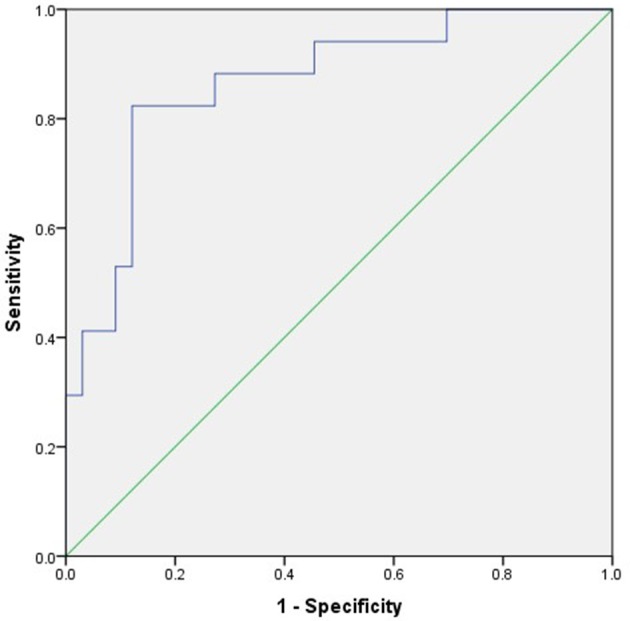
ROC curve of the predictive value of NLR for poor prognosis of AE.

All factors with a *P*-value < 0.20 in Table 2 were included in a multivariate logistic regression model. Multivariate logistic regression analysis showed that NLR (odds ratio [OR] 2.169, 95% confidence interval [CI] 1.029–4.570; *P* < 0.05) was an independent risk factor associated with poor prognosis of AE (Table 3). ROC analysis of NLR to predict poor prognosis of AE showed that the area under the curve was 0.866 (95% CI, 0.759–0.974; *P* < 0.001). Based on the ROC curve, the optimal cutoff value was 4.45 (sensitivity, 0.824; specificity, 0.879; shown in Table 4 and Figure 2).

**Table 3:** Multivariate analysis of factors associated with a poor prognosis.

**Variables**	**OR**	**95% CI**	***P*-value**
Consciousness disorders	11.995	0.173–833.456	0.251
Epileptic seizures	1.003	0.31–32.757	0.999
Extrapyramidal symptoms	10.157	0.529–195.094	0.124
EEG results	18.206	0.209–1586.043	0.203
Brain MRI results	1.189	0.53–26.628	0.913
Pulmonary infection complications	1.071	0.029–40.049	0.970
Albumin	1.792	0.100–32.115	0.692
Neutrophil count	0.089	0.002–3.640	0.201
Lymphocyte count	6.918	0.059–812.704	0.426
NLR	2.169	1.029–4.570	0.042

*OR, odds ratio; CI, confidence interval; EEG, electroencephalogram; MRI, magnetic resonance imaging; NLR, neutrophil-to-lymphocyte ratio*.

**Table 4:** Receiver operating characteristic curve-related statistical indicators.

**Prediction**	**AUC**	**95% CI**	***P***
NLR	0.866	0.759–0.974	< 0.001

*NLR, neutrophil-to-lymphocyte ratio; AUC, area under the curve; CI, confidence interval*.

**Figure 3:**
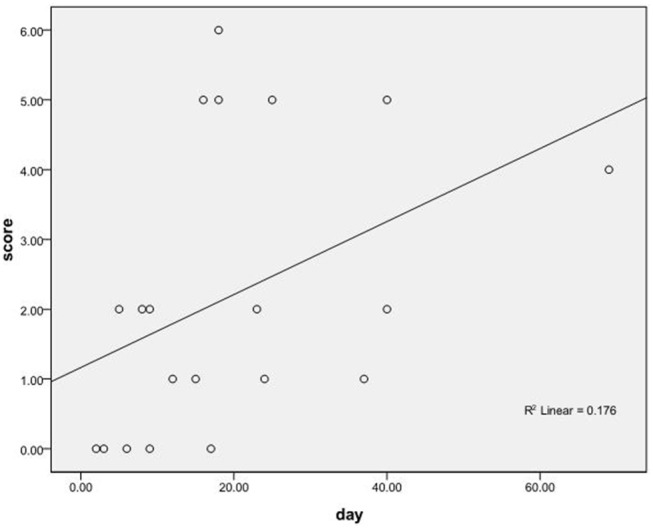
The correlation between the immunotherapy latency and the prognostic mRS scores of 20 patients who received immunotherapy.

The Spearman rank correlation test was performed to analyze the correlation between the immunotherapy latency and prognostic mRS scores of 20 patients who received immunotherapy. There was a positive correlation between the immunotherapy latency and mRS score (r_s_ = 0.535, *P* < 0.05; Figure 3).

## Discussion

In this study, we retrospectively analyzed patients with an initial diagnosis of AE. We focused on clinical features, laboratory and imaging examinations, and EEG findings; moreover, we evaluated which factors are related to a poor prognosis. This study revealed that an increase in NLR was an independent risk factor for predicting the poor prognosis of AE. Prior to our study, the role of NLR in AE had not been examined, and this study presented a novel finding to predict the poor prognosis of AE.

AE is an increasingly recognized immune-mediated brain disease (10). This disease includes a heterogeneous group of encephalitic syndromes, which is divided into the following categories: new-type AE associated with antibodies to neural surface antigens and classic paraneoplastic limbic encephalitis (LE) associated with onconeural antibodies against intracellular antigens (25). It is reported that cases with surface antigen antibodies present a different immune reaction than that of cases with intracellular antigen antibodies. T cells are thought to play a cytotoxic role in cases with intracellular antigen antibodies (26), whereas antibody and/or complement-mediated mechanisms are considered to be responsible for neurodegeneration in encephalitis with surface antigen antibodies (10). Chronic inflammation, which is triggered by the overproduction of autoantibodies, inflammatory cytokine release, and deposition of the immune complex, plays an important role in the disease development process of AE (25). Abnormal immune regulation and persistent inflammation are critical pathological manifestations in the disease development process of AE.

NLR has been suggested as an indicator of systemic inflammation (11, 27). Compared with independent neutrophils, lymphocytes, and total white blood cell counts, NLR is less affected by various physiological and pathological conditions. NLR is an inexpensive, easily measurable, and widely available blood test affected by both innate immune response (mediated by neutrophils) and adaptive immune response (mediated by lymphocytes) (20). Changes in NLR may reflect the shifting balance between inflammatory activity and immune activity (28). Inflammation is a response to acute or chronic tissue damage caused by infection, ischemic injury, physical injury, and other types of trauma. When these conditions occur, the immune system will lead neutrophils, lymphocytes and other inflammatory cells to accumulate in the site of damage (14). Under inflammatory conditions, neutrophil and lymphocyte counts present temporary changes. High levels of neutrophil infiltration may result from cytotoxicity in response to changes in the balance of pro-inflammatory and anti-inflammatory cytokines (29). The reason why NLR can predict prognosis may be summarized in two aspects: neutrophils are associated with a much quicker response, while lymphocytes are involved with more adaptive, chronic responses of the immune system (30). In the process of inflammation and immunity, neutrophils can destroy tissue directly by producing the enzyme myeloperoxidase and free radicals, and regulating the activity of other cell types (31). Moreover, some treatments such as immunotherapy can cause changes in NLR. Therefore, the routine blood results in our study were recorded within 24 h of admission to avoid interference from immunotherapy.

As an indicator of systemic inflammation, NLR has been frequently used to predict outcomes in many diseases. Prior studies have shown that altered NLR is related to decreased overall survival (OS) in various cancers. For example, Ma et al. detected that NLR is a significant predictor for recurrence in stage III melanoma patients (32). Shimada et al. suggested a high preoperative NLR as a biomarker to identify patients with a poor prognosis after resection for primary gastric cancer (33). Azab et al. found that NLR level >3.3 is an independent significant predictor of mortality in patients with breast cancer (34). Some studies have also reported that increased NLR is associated with higher rates of mortality in patients with acute heart failure or acute coronary syndrome (31, 35). In addition, a high NLR is also associated with a risk of death in critically ill patients, including patients with severe sepsis or septic shock (27, 36). Kim et al. demonstrated that NLR is a stronger independent predictor of postoperative acute kidney injury (AKI) (37). Another retrospective study of prognostic factors in patients with acute respiratory distress syndrome (ARDS) suggested that a high NLR (>14) independently predicts a poor prognosis in patients with ARDS (38). Based on recent studies, NLR is increased in patients with autoimmune diseases. In a previous study on the relationship between NLR and systemic lupus erythematosus (SLE), a high NLR was independently associated with SLE (39). In a meta-analysis on the relationship between hematological indices and autoimmune rheumatic diseases (ARDs), including ankylosing spondylitis (AS), Behçet's disease (BD), and rheumatoid arthritis (RA), NLR was recommended as a diagnostic biomarker for ARDs (22). Our study results extended previous reports on the prognostic role of NLR.

In fact, in clinical work, antibody-positive AEs are the minority, while most AEs are probable AEs or possible AEs. Several previous studies on prognostic factors of AE also evaluated different AEs, including “definite” and “probable” AE cases, in the same study (2, 40). In our study, among patients who received antibody testing, the proportion of patients with a definite diagnosis of AE (36%) was in the range reported in the literature (2, 40, 41). AE can appear as several different syndromes, classically presenting with decreased levels of consciousness (symptoms progress over a period of days or weeks) that eventually develops into coma (42). Extrapyramidal symptoms, such as dystonic seizures, chorea, or abnormal posture of the limbs, occur with anti-NMDAR encephalitis. In adults with anti-NMDAR encephalitis, facial, and limb writhing movements may be most notable in the comatose phases of the disease (43). In our data, 71% (5/7) of patients with anti-NMDAR encephalitis developed extrapyramidal symptoms. Seizures are common in AE and may occur at any stage of the disease, and studies have revealed that status epilepticus can predict a poor outcome for encephalitis (44, 45). Several studies on the death factors of encephalitis in the ICU have shown that status epilepticus, central hypoventilation, and complications (such as multiple organ dysfunction or severe pulmonary infection) are predictors of poor prognosis of encephalitis (44, 46, 47). However, in our study, consciousness disorders, epileptic seizures, extrapyramidal symptoms, and pulmonary infection complications were associated with adverse outcomes but were not independent predictors of poor prognosis. This result may be attributed to the following reason. With the development of diagnostic techniques and the availability of effective treatments, the predictors of poor prognosis may change. For example, a retrospective study of anti-NMDAR encephalitis also found that disturbance of consciousness, central hypoventilation, and complications are not independent predictors of poor prognosis (48). Another French study reported that status epilepticus in patients with anti-NMDA receptor encephalitis is unrelated to poor prognosis (49). Our results were essentially consistent with the results of previous related studies.

Serum albumin has been suggested as a prognostic factor in various diseases, including Guillain-Barre syndrome (GBS) (50). Jang et al. reported that low albumin levels are a significant indicator of AE prognosis (51). In our study, low albumin was associated with poor prognosis in univariate analysis but not in multivariate logistic regression analysis. This result may be because albumin levels in patients with low albumin have been improved during hospitalization without affecting patients' prognosis.

In most cases of AE, brain MRI shows normal or only non-specific inflammation changes (52). Some abnormal cases may present with increased signal on T2-weighted images, especially in the medial temporal lobe. In our study, abnormal MRI findings were associated with poor prognosis of AE in univariate analysis. The reason for this finding may be related to the anatomy and physiological functions of the involved brain regions. Frontal and temporal lobe lesions can easily lead to psychiatric symptoms and secondary epilepsy seizures; parietal lobe lesions are susceptible to sensory disturbances, and basal ganglia lesions are prone to causing extrapyramidal symptoms or paralysis, among other nervous system sequelae. EEG often exhibits focal or diffuse slow-wave activity associated with one or more epileptic foci in all types of AE. In addition to what may be called an “extreme triangle brush” pattern in patients with anti-NMDAR encephalitis, there are no characteristic EEG abnormalities for other forms of AE (53). However, in the acute phase of encephalitis, aggravation of slow-wave activity is often accompanied by disturbance of consciousness, indicating that the injury is severe. Some studies have reported that EEG can predict prognosis in autoimmune or infective encephalitis, and normal EEG is a predictor of good prognosis (54). In our study, abnormal EEG was associated with poor prognosis of AE in univariate analysis. This study demonstrated that inflammatory changes in CSF are not related to prognosis. Although some patients with AE have moderately increased CSF lymphocytes, a lack of increase in cell numbers does not rule out this diagnosis (52). Most patients with AE have detectable neuronal autoantibodies in the CSF even if the CSF test is normal (8).

Immunotherapy for AE includes first-line therapy (steroids, IVIg, plasma exchange, or all) and second-line therapy (rituximab, cyclophosphamide, or other). Steroids are always the first option. Two weeks or more should be allowed for first-line therapies to work. If the patient remains very ill after first-line treatments, second-line therapy is typically administered (43). In the present study, early initiation of immunotherapy was associated with a good prognosis. Correspondingly, previous studies suggested that early immunotherapy improves the outcome of AE. A multi-institutional observational study of the prognosis of 577 patients with anti-NMDAR encephalitis showed a correlation between early immunotherapy and good prognosis, and it took more than 18 months for patients to recover (55). Another study suggested that patients who received immunotherapy within 40 days of onset had a better outcome than those who started immunotherapy after 40 days of onset (56). Our results were consistent with those of previous studies. Notably, not all patients with AE will respond to immunotherapy, but this does not mean that patients with AE cannot achieve a good outcome without immunotherapy. For example, in our study, two patients with anti-NMDAR encephalitis also achieved a good prognosis without immunotherapy. Therefore, considering the response to immunotherapy as a part of the diagnostic criteria of AE is not unreasonable. The speed of recovery, degree of residual deficit, and frequency of relapse differ greatly in different types of AE (8).

There is no known laboratory marker that predicts the poor prognosis of AE. Our study is the first to investigate the prognostic value of NLR in patients with AE. NLR has the advantage of low economic cost, no damage, and convenience. However, our study has several limitations. First, the present study was a retrospective design, thus, controlling for confounding factors was difficult. Prospective validation of NLR is required. Second, this study was conducted in a single institution, and the sample size of this study was small. Third, other inflammatory biomarkers, such as C-reactive protein (CRP), were not investigated, and the relationship between NLR and other inflammatory biomarkers could not be evaluated. Finally, there is still no consensus on the cutoff values to define the levels of NLR. The optimal cutoff value found in our study was 4.45, which is different from the values used in prior studies. The difference in cutoff points may be due to differences in the study population.

In conclusion, our study found that NLR may have predictive value for the poor outcomes of AE. Prospective validation of NLR is required. In addition, we revealed that early initiation of immunotherapy was associated with a good prognosis.

## Ethics Statement

This study was carried out in accordance with the recommendations of the ethical guidelines of the Declaration of Helsinki with written informed consent from all subjects. All subjects gave written informed consent in accordance with the Declaration of Helsinki.

## Author Contributions

XQ, HZ, CY, and ZX contributed to the conception and design of the work. XQ, HZ, and DL contributed to the acquisition, analysis, and interpretation of the data. HZ, DL, JW, PX, and JZ prepared figures and tables. XQ and HZ contributed to drafting the manuscript. XQ, ZJ, YZ, and CY contributed to statistical analysis. XQ, HZ, ZF, and ZX discussed the results. XQ and ZX revised the manuscript. XQ and HZ contributed equally and share first authorship.

### Conflict of Interest Statement

The authors declare that the research was conducted in the absence of any commercial or financial relationships that could be construed as a potential conflict of interest.
